# Use of Mutated Self-Cleaving 2A Peptides as a Molecular Rheostat to Direct Simultaneous Formation of Membrane and Secreted Anti-HIV Immunoglobulins

**DOI:** 10.1371/journal.pone.0050438

**Published:** 2012-11-28

**Authors:** Kenneth K. Yu, Kiefer Aguilar, Jonathan Tsai, Rachel Galimidi, Priyanthi Gnanapragasam, Lili Yang, David Baltimore

**Affiliations:** 1 California Institute of Technology, Pasadena, California, United States of America; 2 Keck School of Medicine of the University of Southern California, Los Angeles, California, United States of America; Shanghai Medical College, Fudan University, China

## Abstract

In nature, B cells produce surface immunoglobulin and secreted antibody from the same immunoglobulin gene via alternative splicing of the pre-messenger RNA. Here we present a novel system for genetically programming B cells to direct the simultaneous formation of membrane-bound and secreted immunoglobulins that we term a “Molecular Rheostat”, based on the use of mutated “self-cleaving” 2A peptides. The Molecular Rheostat is designed so that the ratio of secreted to membrane-bound immunoglobulins can be controlled by selecting appropriate mutations in the 2A peptide. Lentiviral transgenesis of Molecular Rheostat constructs into B cell lines enables the simultaneous expression of functional b12-based IgM-like BCRs that signal to the cells and mediate the secretion of b12 IgG broadly neutralizing antibodies that can bind and neutralize HIV-1 pseudovirus. We show that these b12-based Molecular Rheostat constructs promote the maturation of EU12 B cells in an *in vitro* model of B lymphopoiesis. The Molecular Rheostat offers a novel tool for genetically manipulating B cell specificity for B-cell based gene therapy.

## Introduction

B cells are responsible for the production of antibodies in response to foreign antigens [1]. The ability to manipulate the antigen specificity of B cells and that of the antibody produced by these cells could be useful for achieving immunization against deadly pathogens such as HIV. In this paper, we describe a novel system for simultaneously expressing IgM-like BCRs and IgG antibody. The system is designed so that the ratio of surface and secreted immunoglobulins can be controlled by appropriate choices of mutations in the 2A peptide. We call this system a “Molecular Rheostat”.

B cells begin their life in the bone marrow as descendants of the more primitive common hematopoietic stem and progenitor cells. As these cells develop into B cells, they undergo sequential RAG1/2-mediated DNA rearrangement of the heavy and light chain immunoglobulin gene loci in a process called V(D)J rearrangement. Cells that successfully complete this process and assemble a functional B cell receptor (BCR) of the IgM isotype on their surface are able to leave the bone marrow to continue further development in the peripheral lymphoid compartments [2], [3]. The generation of the IgM BCR is central to B cell development and function. It is both necessary for the normal development of B cells [4], [5], [6], and sufficient for directing B cell development. In transgenic animals. the provision of a pre-rearranged IgM heavy chain and light chain transgene shuts down the rearrangement of endogenous heavy and light chain genes (allelic exclusion), and guides the ordered development of functional B cells with specificity defined by the transgene [7], [8]. These observations highlight the importance of the IgM BCR in B-cell biology and suggest that any artificial molecule that functions as a BCR would need to mimic IgM for it to be able to direct B-cell development.

The mature B cells patrol the body in the general and lymphatic circulations, using their BCRs as antigen sensors. When a cognate antigen engages the BCR, the B cell becomes activated and enters into a germinal center reaction in the lymph node or spleen in a dance of mutual activation with T cells; this process leads to further development into memory B cells or differentiation into antibody-producing plasma cells. The memory B cells will provide a more rapid and higher quality antibody response in the future when the same antigens are encountered again. The plasma cells produce antibodies against the inciting antigens, which leads to their eventual clearance from the body [1]. As B cells differentiate into plasma cells, they switch from producing the membrane-bound IgM BCR to making a soluble, secreted antibody. The genomic machinery for effecting the switch is complex and involves alternative-splicing of the heavy-chain pre-mRNA [9], [10], [11], [12], [13]. The switch replaces the hydrophobic amino acids that form the trans-membrane anchor with a hydrophilic tail that enables the secretion of the BCR as free antibody. The antibody retains the same specificity and isotype as the BCR.

Initially we attempted to create such a switchable expression system by exploiting the regulated alternative-splicing pathway of the heavy chain locus in B cells. That approach proved to be difficult due to the size of the locus (∼1 Mbp), the challenges of employing RNA alternative splicing in a lentiviral vector context, and the complexity of the natural alternative-splicing system in B cells. Therefore, we sought to develop a simplified, synthetic system that, while not fully switchable, still enables the simultaneous expression of the secreted and membrane-bound BCR at a defined and controllable ratio. This Molecular Rheostat system uses mutant self-cleaving 2A peptides to achieve control over the relative amounts of secreted and membrane-bound immunoglobulins.

2A peptides are “self-cleaving” peptides that are derived from viruses [14], [15]. They are involved in the processing and expression of polyproteins. Mechanistically, these peptides do not really undergo a “self-cleaving” event in the sense of breaking a *pre-existing* peptide bond; rather the presence of the 2A element in the mRNA causes the translating ribosome to undergo an intra-ribosomal, translational termination-and-restart event during the synthesis of the nascent polypeptide chains. The peptide bond between the first and second polypeptide deriving from the same mRNA is in fact not formed during translation. As a result, when these two polypeptides are liberated from the ribosome, they appear as two separate proteins [16], [17], [18]. Because the apparent effect is as if a single polypeptide had been cleaved by an enzyme post-translationally into two separate polypeptides, for consistency with their historic description, we will still refer to 2A peptides as “self-cleaving” peptides, even though in reality they mediate a ribosomal stop-and-restart event, referred to as a “StopGo” action of the 2A element [19].

Several 2A peptides appear to have near 100% cleavage efficiency in their native contexts, but they can be made to cleave at lower efficiencies when they are mutated at key amino acid residues or introduced into non-native sequences [20], [21], [22]. By engineering the peptides with reduced efficiency of cleavage, we show that we can control the relative amounts of co-expressed BCR and antibody by using different mutant 2A peptides. The different choices of the 2A peptides are like the different settings of a rheostat, allowing one to select, or tune, the relative amounts of secreted and membrane-bound protein that the constructs produce. Thus we call the system a “Molecular Rheostat” for controlling gene expression, and the constructs, Molecular Rheostat constructs.

## Materials and Methods

### Constructs

**Figure 1:**
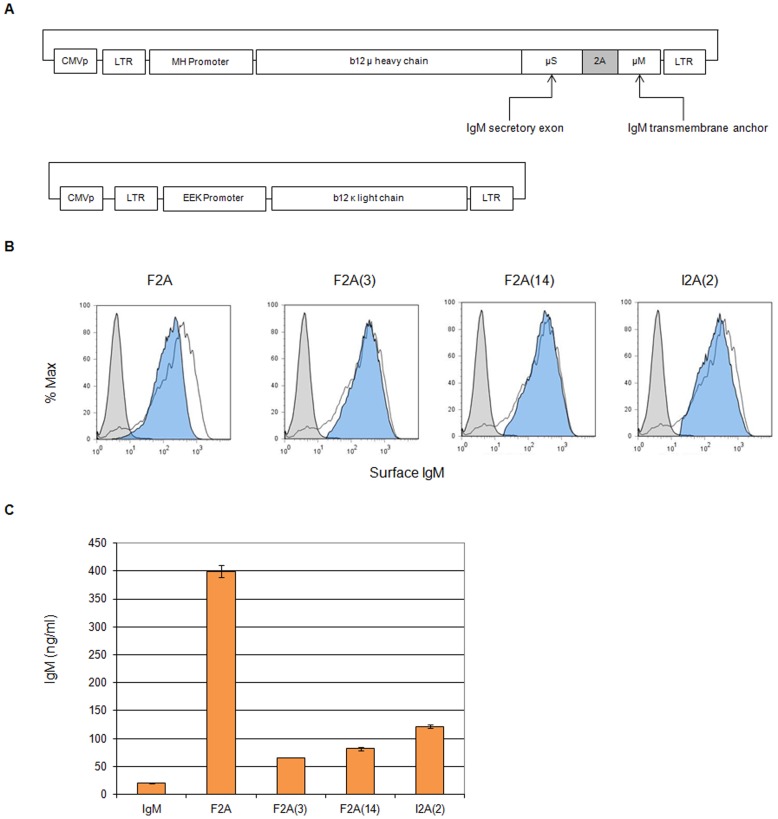
Simultaneous production of IgM-like BCR and secreted IgM using mutant 2A peptides. (A) Schematic representation of the IgM version of the Molecular Rheostat Immunoglobulin constructs. Shown at the top is the design of the heavy chain constructs, containing a secreted IgM heavy chain linked via a 2A element to the transmembrane region (41 aa inclusive of the C-terminus) of the membrane form of the IgM heavy chain. 2A: location of self-cleaving 2A elements. See (B), (C), and Table 1 for the specific 2A peptides and their sequences. Shown at the bottom is the light chain construct, furnishing the b12 κ light chain. CMVp: CMV promoter. LTR: long terminal repeat. MH and EEK promoters: internal B cell specific promoters from human IgM heavy and κ light chain loci, respectively. b12 µ heavy chain: IgM heavy chain with variable region corresponding to that of the b12 broadly neutralizing antibody. (B) IgM surface staining of 293T cells co-transfected with the same amount of the IgM Molecular Rheostat constructs (heavy chain to light chain in 1∶1 ratio by mass) together with a third construct expressing human Igα and Igβ. The cells were harvested 48 hours post-transfection. All IgM Molecular Rheostat constructs produced surface IgM. White: Membrane-bound IgM control. Blue: Molecular Rheostat Constructs. Gray: GFP control. (C) IgM ELISA of supernatants of transfected cells. The constructs expressing different 2A mutants produced different amounts of secreted IgM. IgM: membrane-bound IgM control.

The first-generation IgM Molecular Rheostat constructs required two vectors. The light chain vector was made by cloning a b12 κ light chain into the FEEKW vector. The heavy-chain variable region of the b12 IgG antibody (from Dr. Gary Nabel, NIH) was grafted onto a secretory version of the human IgM gene cloned from a BAC containing a partial human heavy chain locus. The resulting secretory form of the IgM heavy chain was then joined via 2A elements to the IgM trans-membrane anchor (corresponding to the last 41 aa from the C-terminus of the membrane-bound form of the human IgM). These heavy chain genes were then cloned into the FMHW vector. The FEEKW and FMHW vector each contains a human κ light chain and heavy chain promoter, respectively, and were described by Luo et al. previously [23]. See Figure 1A for a schematic.

**Figure 2:**
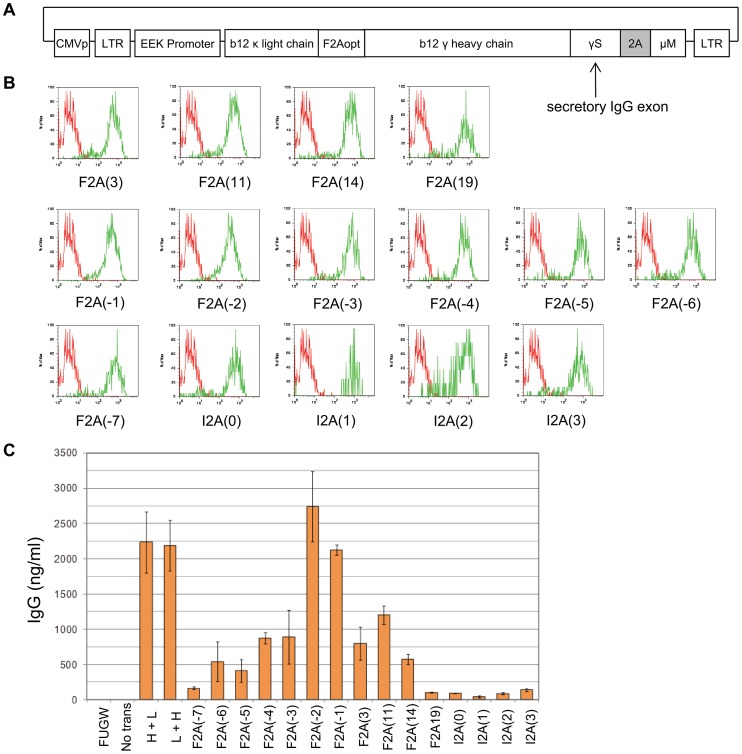
Screening Molecular Rheostat construct library for simultaneous expression of chimeric IgG/M BCR and secreted IgG. (A) Schematic representation of the chimeric b12 IgG/M Molecular Rheostat constructs. The light chain and each of the chimeric heavy chains were combined into single constructs co-expressing the light chain and the chimeric heavy chain. The light and heavy chains are joined by another 2A peptide linker (denoted F2Aopt). 2A: location of mutant self-cleaving 2A elements. See (B), (C), and Table 1 for the specific 2A peptides screened and their sequences. 2Aopt: optimized 2A element with a furin cleavage site at 5′ end. CMVp: CMV promoter. LTR: long terminal repeat. EEK: internal B cell specific promoter. b12 γ heavy chain: IgG heavy chain with the variable region corresponding to that of the b12 broadly neutralizing antibody. (B) Surface staining for human IgG. 293T- Igαβ cells were transfected with the same molar amount of chimeric IgG/M Molecular Rheostat constructs, and analyzed for the expression of surface IgG by flow cytometry. All constructs produced surface-bound chimeric IgG/M BCR detected as human IgG. Green: Molecular Rheostat Constructs. Red: Secretory IgG (L+H) control. (C) IgG ELISA of supernatants of transfected cells. Different Molecular Rheostat constructs produced different amounts of secreted b12 IgG. FUGW: GFP containing vector control. L+H and H+L: secretory b12 IgG controls; H+L has the light chain in the first position and heavy chain in the second position; L+H is in the opposite order.

**Table 1:** Nomenclature and amino acid sequences of different 2A peptides.

2A Mutant	Mutation Type	Amino Acid Sequence
F2A	Wild-type	QLLNFDLLKLAGDVESNPGP
F2A(−1)	1aa N-terminal deletion	LLNFDLLKLAGDVESNPGP
F2A(−2)	2aa N-terminal deletion	LNFDLLKLAGDVESNPGP
F2A(−3)	3aa N-terminal deletion	NFDLLKLAGDVESNPGP
F2A(−4)	4aa N-terminal deletion	FDLLKLAGDVESNPGP
F2A(−5)	5aa N-terminal deletion	DLLKLAGDVESNPGP
F2A(−6)	6aa N-terminal deletion	LLKLAGDVESNPGP
F2A(−7)	7aa N-terminal deletion	LKLAGDVESNPGP
F2A(3)	Point mutation	QLLNFDLLKLAGDV**Q**SNPGP
F2A(11)	Point mutation	QLLNFDLLKLAGDVE**I**NPGP
F2A(14)	Point mutation	QLLNFDLLKLAGDVES**E**PGP
F2A(19)	Point mutation	QLLNFDLLKLAGDVESNP**A**P
I2A(0)	Wild-type	TRAEIEDELIRRGIESNPGP
I2A(1)	Point mutation	TRAEIEDELIR**A**GIESNPGP
I2A(2)	Alternative codon	TRAEIEDELIRRGIESNP**G**P
I2A(3)	Point mutation	TRAEIEDELIRRGIESNP**A**P

The chimeric IgG/M Molecular Rheostat constructs were created by cloning the EEK or MH promoters, the b12 light and heavy chains, the 2A sequences, and an the 3′ region of the human IgM BCR gene corresponding to the last 41 amino acids into either a pHAGE2 or pHAGE6 lentiviral vector. Both are third-generation, self-inactivating lentiviral vector backbones based on the pHRST [24], [25]. See Figure 2A for a schematic.

### Transfections

293T cells were grown to 50–75% confluence on 30 cm dishes and were transfected in 15 ml D10 media (DMEM plus 10% heat-inactivated fetal bovine serum, supplemented with 20 mM L-glutamine, 1000 IU/ml penicillin, and 1000 µg/ml streptomycin, filtered through a 0.22 µm PES membrane bottle-top filter) for 24 h. The transfections used the TransIT-293 reagent (Mirus Bio, Madison WI) or BioT (Bioland Scientific, Paramount CA) according to manufacturer's instructions, using a total of 40 µg DNA.

### Lentiviral Vector Production

293T cells were transfected with lentiviral vectors. After 24 h of incubation, the supernatant was pipetted off the cells and filtered through a 0.22 µm PES membrane bottle-top filter into a collection bottle. 15 ml of fresh D10 media was then filtered through the bottle-top filter into the collection bottle to reduce virus waste from supernatant that the filter absorbed. The collected supernatant was stored at 4°C, and 30 ml of fresh D10 media was added to the dish. This collection process into the same collection bottle was repeated 4 to 5 additional times at 12 h intervals. All of the collected supernatant was centrifuged at 10000 rpm for 12–24 h at 4°C to pellet the virus, and the supernatant was poured off the pellet. The pellet was re-suspended in 500–1000 µL DMEM media (for 293T transductions) or RPMI media 1640 (for OCI-Ly7 or EU12 transductions) and incubated on ice at 4°C for 12 h.

### Lentiviral Transductions

0.5–1×10^6^ 293T, OCI-Ly7, or EU12 cells were suspended in 1 mL of D10 media for 293T transductions or C10 media (RPMI 1640 plus 10% heat-inactivated fetal bovine serum, supplemented with 25 µM β-mercapto-ethanol, 1000 IU/ml penicillin, and 1000 µg/ml streptomycin, filtered through a 0.22 µm PES membrane bottle-top filter) for OCI-Ly7 or EU12 transductions in 12 well plates, and 400–600 µL of virus re-suspensions or dilutions thereof was added to each well. 10 mg/mL polybrene (Millipore, Billerica, MA) was added so that the final polybrene concentration was 10 µg/mL in each well. The transductions were incubated for 24 h before the cells were passaged.

The EU12 cell line was a kind gift from Dr. Zhixin Zhang (University of Nebraska Medical Center, Omaha, NE) and Dr. Max Cooper (Emory University, Atlanta, GA), and was described in detail by Zhang et al. [26]. The OCI-Ly7 B-cell line was kindly provided by Dr. Louis M. Staudt (National Cancer Institute, NIH, Bethesda, MD), and was originally described by Tweeddale et al. [27].

### Cell Line

The 293T-Igαβ cell line was created by infecting 293T cells (purchased from ATCC) with a lentivector carrying the Igα and Igβ genes using the lentiviral transduction procedure described above.

### Tissue Culture

293T and 293T Ig-αβ cells were grown in D10 media. The cells were passaged 1∶5 every other days. OCI-Ly7 and EU12 cells were grown in C10 media. The cells were passaged 1∶5–1∶10 every other day to maintain a density between 10^5^–10^6^ cells/ml.

### Flow Cytometry

For flow cytometric analysis, cells were first washed in PBS with 2% FBS, and then stained with combinations of the following antibodies: anti-human-IgG-APC (BD Pharmingen, San Diego, CA), anti-human-IgG-PE (BD Pharmingen), anti-human-IgM-PE/Cy5 (BD Pharmingen), anti-CD10-PE (Biolegend, San Diego, CA). The cells were then analyzed on a BD FACSCalibur flow cytometer.

### Cell Sorting

Cells were prepared as in flow cytometric analysis and were sorted with the assistance of Sylvia Chavira at the University of Southern California’s Clinical Pathology Laboratory using a MoFlo FACS cell sorter.

### Calcium Flux Assay

Calcium flux measurements were made using the protocol given by Bondada *et. al.* [29], with the following modifications: cells were washed, pelleted, and resuspended in Dye Loading Buffer (HBSS with Ca^2+^ and Mg^2+^ plus 4% 100mM probenecid, 2% 1 M HEPES buffer, and 1% heat-inactivated fetal bovine serum) and were incubated with 4 µg/mL Fluo-3 AM and 1 µg/mL FuraRed AM dyes in the presence of 0.02% (w/v) pluronic F-127 for 30 m. The cells were again washed, pelleted, and resuspended in Dye Loading Buffer and were kept at room temperature until they were analyzed on a BD FACSCalibur flow cytometer equipped with a circulating 37°C water bath on the sample port. During analysis, cells were stimulated with goat F(ab’)_2_ anti-human IgG γ F_c_-specific antibodies (Invitrogen, Carlsbad, CA) or with goat F(ab’)_2_ anti-human IgM µ F_c_-specific antibodies (Southern Biotech, Birmingham, AL) and a ratiometric measurement between the Fluo-3 AM and FuraRed AM dye channels was made for 512 s. On some samples, ionomycin controls were performed to calibrate the dynamic signaling range.

### ELISA

Supernatants from cultured cells were analyzed using Human IgG ELISA Quantitation Set (Bethyl Laboratories, Montgomery, TX) according to manufacturer’s instructions.

### Surface Plasmon Resonance gp120 Binding Assay

The Surface Plasmon Resonance (SPR) gp120-binding assays were performed as previously described by Klein et al. [28]., with the following modifications: All experiments were done in-house. b12 antibody supernatants were produced from transfection of 293T cells.

### *In Vitro* Neutralization Assay

*In vitro* neutralization assays were performed as previously described by West *et. al*. [30], with the following modifications: All experiments were done in-house. Pseudoviruses were produced by co-transfecting HEK293T cells with an Env SF162 expression plasmid and a replication-defective backbone plasmid, PSG3minusEnv. Each mutant F_c_ and unmodified fragment version of b12 samples was tested in duplicates.

## Results

### IgM Molecular Rheostat Immunoglobulin Genes Mediate Co-Expression of IgM-Like BCR and Secreted IgM Antibody

As a pilot experiment to test whether the mutated 2A peptides can mediate co-expression of surface and secreted immunoglobulins, we constructed the first-generation of Molecular Rheostat Immunoglobulin genes by joining the secreted version of the b12 IgM heavy chain to the transmembrane domain of the IgM BCR via a mutated 2A peptide. The transmembrane domain is defined as the M1 and M2 exons from the human IgM locus and comprises the last 41 amino acids of the membrane bound IgM BCR (Figure 1A). We call these “IgM Molecular Rheostat” constructs. We chose the wild type F2A and two mutant peptides as well as another F2A-like element derived from a silk-worm virus, based on previous work by Donnelly et al. [20], in which they observed reduced cleavage efficiencies when certain mutations are introduced. The four mutants we chose are designated F2A, F2A(3), F2A(14), and I2A(2). See Table 1 for the nomenclature and the amino acid sequence for each of the 2A elements.

We cloned these IgM Molecular Rheostat genes into a lentiviral vector plasmid (FMHW), which also doubles as a mammalian expression vector under the control of a CMV promoter (Figure 1A). We co-transfected these heavy chain vectors together with a separate vector carrying the b12 light chain (FEEKW-b12L) and a mammalian expression vector carrying the human Igα and Igβ genes (phIgαβ) into 293T cells. The FMHW vector backbone and the related FEEKW were previously described by Luo et al. [23]. Briefly, both are lentiviral vectors that contain promoters derived from B-cell-specific genes. The FMHW vector carries the MH promoter, which is composed of a human immunoglobulin heavy chain variable region promoter coupled to the µ-intronic enhancer. The FEEKW vector carries the EEK promoter, which is composed of the human κ light chain promoter coupled to κ light chain enhancer elements. We analyzed the transfected cells and their supernatants by flow cytometry and human IgM ELISA 48 hours later. All transfected cells showed surface expression of the IgM Molecular Rheostat BCR and secreted IgM into their supernatants (Figure 1B and 1C).

### Chimeric IgG/M Molecular Rheostat Constructs Mediate Simultaneous Expression of chimeric IgG/M BCRs and Secreted IgG Antibody

We next attempted to adapt the Molecular Rheostat format to the production of an IgG antibody in an effort to mimic an isotype-switched secretory IgG while preserving the signaling properties of an IgM, which is required for normal B cell development. Furthermore, we wished to explore whether we could manipulate the ratio of surface-bound to secreted immunoglobulins by making appropriate mutations in the 2A elements. To test these ideas, we constructed a library of chimeric IgG/M Molecular Rheostat Immunoglobulin genes, in which a complete secretory b12 IgG is joined to the transmembrane anchor and the intracellular domain of the IgM BCR via different 2A peptides (Figure 2A). The library includes 2A peptides listed in Table 1.

To reduce the number of vectors that need to be transfected and anticipating the need to use the vectors in the context of lentiviral transduction, where it would be advantageous to work with a single vector, we fused the b12 κ light chain with the Molecular Rheostat heavy chain transgene by joining the b12 κ light chain to the b12 IgG heavy chain via a different F2A element, F2Aopt. F2Aopt is codon-optimized for human expression and contains a furin cleavage site before the 2A element.

Additionally, to ensure consistency of Igα and Igβ expression across the cells used to test the Molecular Rheostat constructs and reduce the number of vectors that need to be transfected, we engineered 293T cells that express human Igα and Igβ by repeatedly co-infecting 293T cells with two lentiviral vectors, FUW-Igα and FUW- Igβ, which carry the Igα and Igβ transgenes, respectively, under the control of a ubiquitin C promoter. The resulting cells are denoted 293T-Igαβ cells.

We transfected the library of chimeric IgG/M Molecular Rheostat constructs into the 293T-Igαβ cells, and 48 hours later analyzed the cells and their supernatants for surface IgG by flow cytometry and secreted IgG by ELISA, respectively. All transfected cells showed surface expression of the IgG/M Molecular Rheostat BCR (detected as surface IgG because the extracellular portion of the chimeric BCR is made up of the heavy chain of IgG) and secreted IgG into the culture supernatant (Figure 2B and 2C). Significantly, while the surface expression of the IgG/M Molecular Rheostat BCR appears comparable across all constructs, there is a range of levels of secreted IgG. This suggests that the different Molecular Rheostat constructs could be used to produce a range of ratios of surface to secreted immunoglobulins by judicious choices of the 2A peptide mutants.

### Chimeric IgG/M Molecular Rheostat Constructs Mediate Expression of a Range of Ratios of Surface BCR to Secretory IgG in the Human B-Cell Line OCI-Ly7

**Figure 3:**
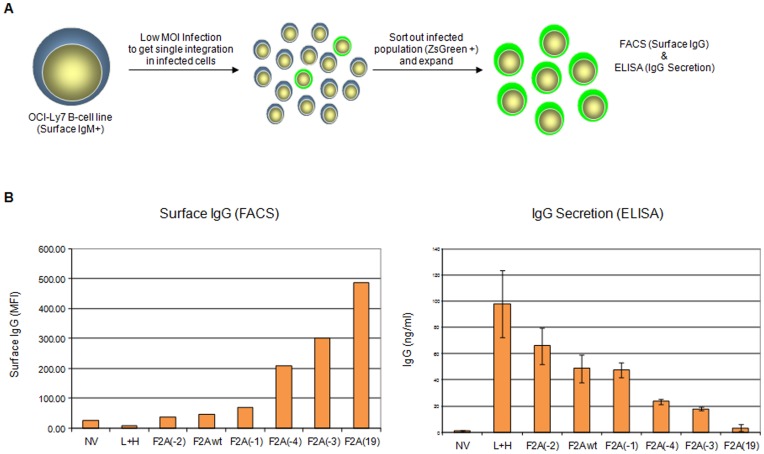
Inversely-related expression of b12 chimeric IgG/M BCR and secreted IgG mediated by mutant 2A peptides. (A) Experimental design for measuring the expression of surface to secreted immunoglobulins by IgG Molecular Rheostat constructs. To establish that it is possible to tune the ratio of surface to secreted immunoglobulins by making appropriate choice of mutations in the 2A peptide, select b12 IgG/M chimeric constructs (based on the experiment with the 293T cells in Figure 2) were modified to include an additional ZsGreen fluorescent protein gene driven by an IRES 3′ of the heavy chain. OCI-Ly7 B cells were infected with at low MOI (∼ 0.1) with this library of constructs and the cells that express the ZsGreen gene were sorted out by FACS. The cells were cultured for 24 hours post-sorting; the cells were analyzed by flow cytometry and supernatants were taken for IgG ELISA. (B) Inverse relationship between surface expression of chimeric b12 IgG/M Molecular Rheostat BCRs and secreted b12 IgG in the supernatants of sorted cells. NV: untransduced control. L+H: secretion only b12 control. The Molecular Rheostat constructs are denoted by the mutant 2A elements they contain.

To validate the results that the chimeric IgG/M Molecular Rheostat constructs can mediate a range of expression ratios of surface BCR to secreted antibodies in human B cells, we used lentiviral vectors to deliver the constructs into the OCI-Ly7 B- cell line, which expresses an endogenous IgM BCR on its surface and therefore should possess the necessary machinery (such as Igα and Igβ co-receptors) for BCR surface expression. To provide an independent marker of lentiviral transduction other than the expression of the Molecular Rheostat Immunoglobulins, we constructed a lentiviral vector, pHAGE2-EEK-IRES-ZsGreen, which contains an Internal Ribosomal Entry Site (IRES) driving a ZsGreen fluorescent protein gene. Based on the results in Figure 2B, we selected six of the IgG/M Molecular Rheostat genes and cloned them into the first position (before the IRES-ZsGreen) of the pHAGE2-EEK-IRES-ZsGreen vector. We then infected OCI-Ly7 cells with the chimeric IgG/M Molecular Rheostat vectors at low MOI (∼ 0.1) to ensure that nearly every cell that was infected had at most one copy of the transgene (Figure 3A). 48 hours after infection, we sorted out the ZsGreen positive cells and allowed these cells to expand for another 48 hours. The cells and supernatants were analyzed by flow cytometry and ELISA, respectively (Figure 3B, left and right panels, respectively). The different mutants produced a range of ratios of surface to secreted immunoglobulins. Significantly, there is an inverse relationship between the amounts of chimeric IgG/M Molecular Rheostat BCR expressed on the surface of the cells vs. the amounts of IgG antibody that was detected in the supernatants, indicating that the mutant 2A elements could be used like a “rheostat”, tuning the ratios of surface to secreted immunoglobulins. This inverse relationship is visualized by plotting the MFI (mean fluorescence intensity) of surface IgG staining in the left panel and contrasting this trend with the levels of secreted antibody production in the right panel of Figure 3B. (See Figure S1 for original FACS histograms of surface IgG staining.) Biasing the Molecular Rheostat towards producing more surface receptors results in a decrease in antibody secretion. Also notably, the rank order of the relative amounts of surface BCR to secreted immunoglobulin expression recapitulates what was observed from the transfection experiment with 293T-Igαβ cells (see Figure 2B and C). For example, from Figure 2B and C, F2A(-2) would be expected to make more secreted IgG than F2A(-4), and this was indeed the case when the constructs were expressed in the OCI-Ly7 B cell line as shown in Figure 3B and C. Furthermore, F2A(-2) made less surface Molecular Rheostat BCR than F2A(-4), as would be expected if the F2A(-2) peptide mediated more efficient cleavage than the F2A(-4) peptide. The library of mutant constructs together constitutes a Molecular Rheostat that we can use to direct tunable ratios of expression of surface BCR vs. secreted immunoglobulin.

### IgG/M Molecular Rheostat Constructs Produce Functional b12 IgG/M Chimeric BCRs are Signaling Competent and Bind to HIV gp120

**Figure 4:**
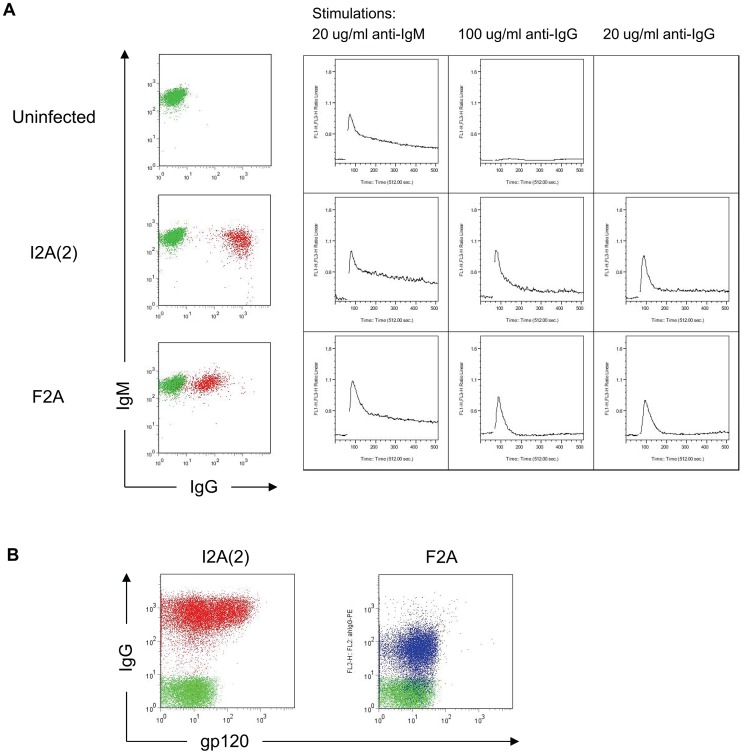
Molecular Rheostat constructs generate functional chimeric IgG/M BCRs that signal and bind to HIV gp120. (A) To test whether the chimeric b12 IgG/M BCRs were functional, the cells were stimulated in a ratiometric calcium flux assay under different stimulation conditions. OCI-Ly7 B cells were infected with a library of chimeric b12 IgG/M Molecular Rheostat constructs that did not contain the IRES-ZsGreen marker gene. 48 hours after infection, cells expressing high amounts of surface IgG by flow cytometry (top 5%) were sorted out. The sorted cells were allowed to rest for 24 hours before anti-BCR stimulation. First column: response of endogenous IgM BCR to anti-IgM stimulation. Second column: high dose (100 ug/ml) anti-IgG stimulation. Third column: low dose (20 ug/ml) anti-IgG stimulation. The BCR expression profiles of the cells at the time of stimulation are shown in the left-most column: endogenous IgM expression (vertical) vs. surface IgG staining from chimeric IgG/M Molecular Rheostat BCR (horizontal). Red: sorted cells expressing the Molecular Rheostat Immunoglobulins. Green: uninfected control cells. The results of anti-IgG stimulations are shown as a panel on the right. First column: response of endogenous IgM BCR to anti-IgM stimulation. Second column: high dose (100 ug/ml) anti-IgG stimulation. Third column: low dose (20 ug/ml) anti-IgG stimulation. (B) Anti-IgG and gp120_MN_ labeling of sorted cells. Red and Blue: I2A(2) and F2A Molecular Rheostat Immunoglobulin vector transduced cells, respectively. Green: untransduced control cells.

To test whether the chimeric IgG/M BCRs were functional, we developed a ratiometric Fluo-3/FuraRed calcium flux assay in which anti-BCR crosslinking antibodies are used to examine whether the BCRs are able to signal in the OCI-Ly7 B cells. We chose two of the 2A peptides from the library, F2A, which cleaves with high efficiency, and I2A(2), which does not cleave well. As the ZsGreen protein interferes with the Fluo-3 calcium-sensitive dye used in the assay, we cloned those two chimeric IgG/M Molecular Rheostat genes into lentiviral vectors that do not have the IRES-ZsGreen marker gene. Lentiviral infections of OCI-Ly7 B cells with these vectors resulted in a variegated pattern of expression of the BCRs. The vector containing the I2A(2) element showed generally higher levels of surface BCR expression than F2A, as expected. While both populations responded to BCR stimulation using a control anti-IgM antibody (Southern Biotech, Birmingham, AB) and an anti-IgG antibody (Sigma, St Louis, MO), the responses were detectable but modest (data not shown). We believe the modest response was due to the effect of averaging the calcium signals over the large range of surface expressions. To ensure we have more homogenous populations for use in BCR stimulations, we FACS sorted out the top 10% of IgG positive cells from each of the populations (Figure 4B), and performed the calcium flux assays on the sorted cells. The cells responded robustly to anti-BCR stimulation (Figure 4A), with a dose-response correlating with the levels of surface IgG/M Molecular Rheostat BCR expression and the concentrations of anti-Ig used. The higher anti-IgG dose (100 ug/ml) gave a stronger calcium signal than the lower dose (20 ug/ml); the cells with more surface Molecular Rheostat BCR expression also generated a stronger and more lasting response.

Additionally, to see whether the chimeric IgG/M BCR would bind to HIV antigens, we co-stained the sorted OCI-Ly7 cells with fluorescently labeled HIV gp120_MN_ and anti-IgG, which respectively interact with the gp120-antigen-binding site of b12 and the γ heavy chain constant region of b12 IgG (Figure 4C). We found that the Molecular Rheostat BCRs on the cells bound to HIV gp120.

### Chimeric IgG/M Molecular Rheostat Constructs Produce b12 IgG Antibody that Neutralizes HIV Pseudovirus with Same Potency as Unmodified b12 IgG

**Figure 5:**
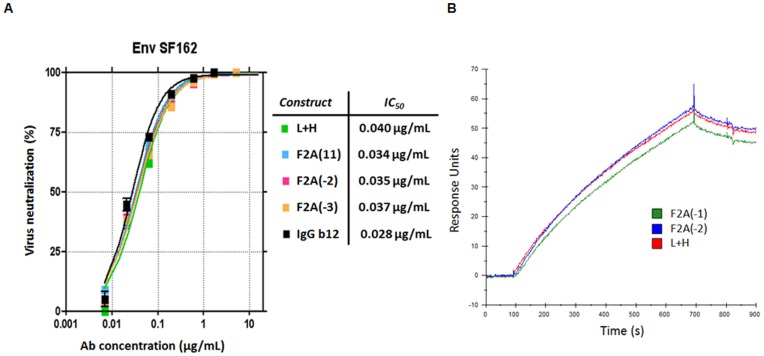
b12 IgG produced by Molecular Rheostat constructs neutralized Env SF162 pseudovirus and bound to GP120. (A) *In vitro* neutralization assay against Env SF162 pseudovirus. The chimeric IgG/M Molecular Rheostat constructs were transfected into 293T cells and IgG in the supernatants were purified using an affinity column. The purified IgG were used in the neutralization assay. The neutralization curves are nearly identical for all mutant 2A constructs and the control b12 IgG (L+H). The IC_50_ values are indicated to the right of the graph. IgG b12: a batch of previously purified b12 IgG included as a positive control for the assay. (B) Surface plasmon resonance (SPR) GP120 binding assay. Supernatants from transfected 293T cells were diluted with media to the same IgG concentration and used in the SPR binding assay. The plot shows nearly identical SPR traces for each of the two tested 2A mutant constructs and the control (L+H).

To determine whether secreted b12 IgG from the Molecular Rheostat system can neutralize infectious virus, we performed an *in vitro* pseudovirus neutralization assay using an Env SF162 pseudotyped HIV-1 pseudovirus on the TMZ-b1 reporter cell line with supernatants from 293T cells transfected with several different chimeric IgG/M Molecular Rheostat constructs according to a protocol previously described by Klein et al. [28]. The neutralization curves demonstrated that secreted Molecular Rheostat b12 IgG antibodies neutralized the Env SF162 pseudovirus as potently as the control b12 IgG antibody (L+H), with IC_50_ values nearly identical to that of the control b12 IgG (Figure 5A). We also performed a surface-plasmon resonance gp120-binding assay. The antibodies tested bound gp120 as well as the control b12 IgG antibody, consistent with the neutralization assay results (Figure 5B).

### Expression of Chimeric IgG/M Molecular Rheostat Immunoglobulins Promote Maturation of EU12 Cells in an *In Vitro* Model of B Cell Development

The promotion of B cell development is one of the major functions performed by the IgM BCR. It thus offers a stringent test of BCR function. To test whether the chimeric IgG/M Molecular Rheostat Immunoglobulin BCR can direct B cell development, we adopted a model of human B cell development using the EU12 cell system [26], [29]. EU12 cells are derived from a B cell leukemia patient; they are uniformly CD19^+^ but exist in a spectrum of primitive (CD34^+^ and CD10^−^, or CD34^+^ and CD10^+^) to more mature (CD34^−^ and CD10^+^, or CD34^−^ and CD10^−^) states. These cells lack a functional BCR, but rarely an IgM BCR is generated spontaneously and the cells proceed to acquire a more mature phenotype.

**Figure 6:**
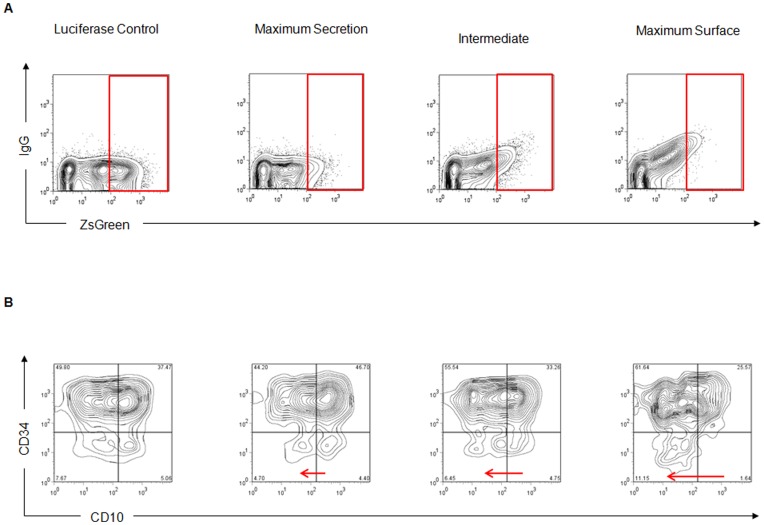
Molecular Rheostat IgG/M BCRs promote downregulation of CD10 in EU12 cells. (A) CD34^+^ EU12 cells (early B cells) transduced with IRES-driven ZsGreen expressing Molecular Rheostat constructs were analyzed by flow cytometry. Surface BCR levels correlate with ZsGreen intensity. Cells transduced with Molecular Rheostat constructs tuned for higher surface expression showed more surface BCR expression with the same ZsGreen expression. The red box shows ZsGreen gating. (B) Gating on high ZsGreen expression, CD10 and CD34 expression was analyzed. Cells transduced with constructs that express higher surface IgG/M BCR levels show a greater extent of CD10 downregulation, suggesting that the Molecular Rheostat BCRs are signaling to the cells and promoting maturation.

We isolated early-stage, CD34^+^ EU12 cells by FACS sorting. These cells were then transduced with lentiviral vectors carrying chimeric IgG/M Molecular Rheostat constructs that give rise to respectively low, intermediate, and high surface BCR expression. A luciferase-carrying vector was used as a control. The cells were allowed to expand, and 4 weeks after transduction the surface expression of chimeric IgG/M Molecular Rheostat BCR and maturation markers were analyzed by FACS (Figure 6). The EU12 cells transduced with Molecular Rheostat constructs tuned at different levels of surface BCR vs. secreted antibody expression showed the expected levels of surface BCR expression (F2A was used for maximum secretion; F2A(11) for intermediate; F2A(19) for maximal surface). Using ZsGreen as a measure of the amount of gene expression from the entire cassette in each cell, the level of chimeric IgG/M BCR expression correlated with the ZsGreen expression level for each of the three Molecular Rheostat constructs (Figure 6A). Gating on the highly expressing cells, we analyzed CD34 and CD10 expression by FACS. We found that the cells that had been transduced with Molecular Rheostat constructs chosen for higher surface BCR expression and less secreted antibody had larger populations of cells that down-regulated CD10 (Figure 6B). This provides further evidence that the chimeric IgG/M BCRs were functional BCRs and were able to promote maturation of B lineage cells.

## Discussion

**Figure 7:**
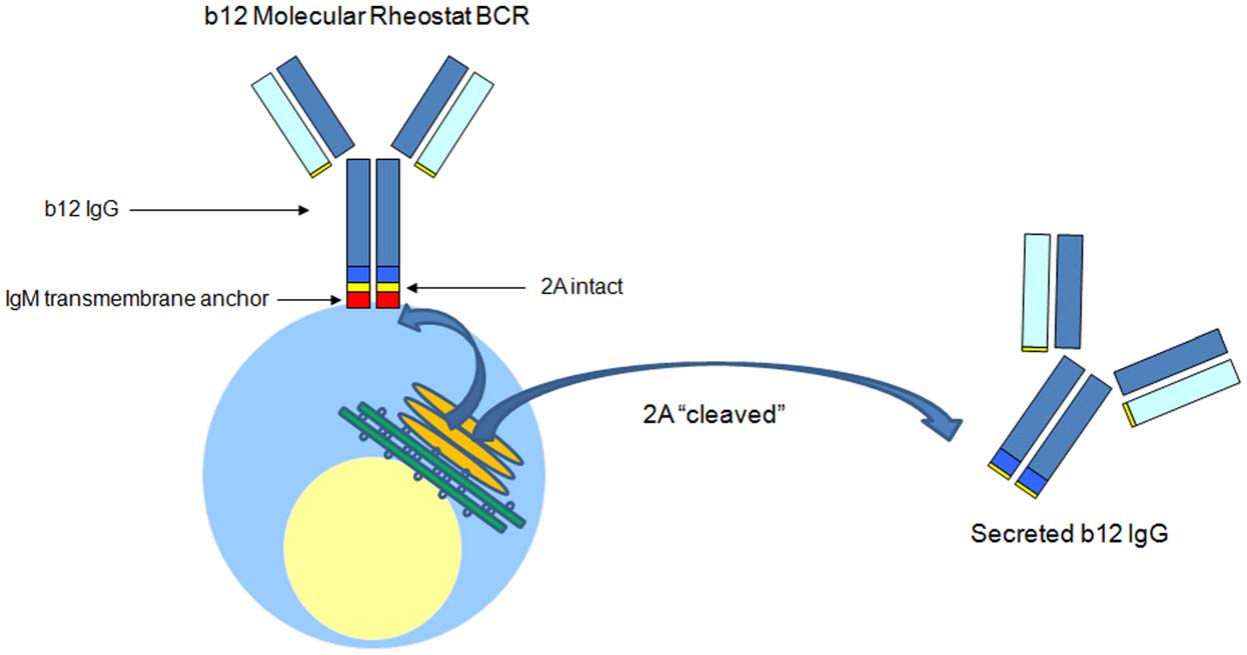
A model of how the b12 IgG Molecular Rheostat Immunoglobulin system directs tunable simultaneous formation of surface BCR and secreted IgG. By making an appropriate choice of the 2A peptide from the library, a specific ratio of secreted IgG and surface chimeric IgG/M BCR can be achieved.

To provide a compact system for genetically manipulating the BCR and antibody specificity of B cells with a lentiviral vector– with the ultimate goal of B cell gene therapy– we created the Molecular Rheostat system. With this system, we could direct various ratios of simultaneous formation of membrane-bound and secreted immunoglobulins by using appropriate mutant 2A “self-cleaving” peptides. We term this a “Molecular Rheostat” because it can be tuned by the choice of mutant 2A (Figure 7). This system provides a synthetic approximation to the process of switching through the evolved RNA alternative splicing mechanism, the route naturally used to make membrane and secreted immunoglobulins in B cells. That process is incompletely understood but appears to require a longer sequence than could be put into a lentiviral construct [9], [10], [12], [13], [30], [31], [32], [33], [34], [35]. By fusing an IgG to the membrane anchor of IgM through different mutant 2A peptides, we constructed a library of chimeric IgG/M Molecular Rheostat constructs and showed that the library of constructs produced both membrane-bound BCR and secreted antibody at controllable ratios. We also showed that the surface chimeric IgG/M Molecular Rheostat BCRs thus produced signaled to B cells and that these BCRs bound to HIV gp120 antigens. We showed that the secreted version of b12 IgG produced by these constructs also bound gp120 and neutralized HIV-1 pseudovirus equally as well as unmodified b12 IgG. Finally, we provided evidence suggesting that the chimeric BCR produced by the Molecular Rheostat system can direct maturation of B cells using a cell line model of B cell maturation. In EU12 cells transduced with vectors carrying the Molecular Rheostat Immunoglobulins, we observed downregulation of CD10, a sign of the maturation of the progenitor cells upon the expression of the chimeric IgG/M Molecular Rheostat BCRs. We note that this effect was dose dependent, with greater size of the CD10^−/^CD34^−^ population being observed in the cells that received the more surface-biased Molecular Rheostat constructs. This suggested that the chimeric IgG/M Molecular Rheostat BCRs were capable of directing the maturation of these cells.

The development of a vector system for programming the development of functional anti-HIV B cells from the hematopoietic stem cells (HSCs) provided the original impetus for our work. We envisioned transducing the HSCs with the lentiviral vector and then transplanting the transduced HSCs back into at risk individuals. The anti-HIV B cells that developed could then serve as a life-long prophylaxis against HIV infection. While one might imagine building such a vector for programming B cells by combining the entire heavy chain and light chain loci into a single vector, the heavy chain locus alone is ∼ 1 Mb, too big to incorporate into a lentiviral vector, which has a insert-carrying capacity of ∼ 10 kb, beyond which titers become too low to be of practical use [36]. We attempted to reduce the size of the vector “payload” by removing the introns of the heavy chain locus, except for the one required for the alternative splicing of the secreted and transmembrane exons, but were not successful because the resulting constructs did not splice correctly. We thus created the Molecular Rheostat system to approximate the natural system, incorporating the additional feature of expressing isotype-switched IgG antibodies while maintaining the signaling properties of an IgM transmembrane domain. Our results, presented in this paper, suggest that the Molecular Rheostat system, which is small enough to be introduced into cells with a lentiviral vector, could be used to direct the *in vivo* maturation of anti-HIV B cells. To see whether this approach could be used as prophylaxis against HIV infections, a detailed characterization in animal models would be needed.

The Molecular Rheostat system coupled with a B-cell specific promoter (such as EEK or MH) can be used as a “gene therapy vaccine” to direct *in vivo* development of an anti-HIV immune system to produce broadly neutralizing anti-HIV antibodies. An attempt to investigate gene therapy as prophylaxis against HIV by vector-mediated delivery of broadly neutralizing antibody to HSCs in a humanized mouse model was previously made by Joseph et al. [37]. However, the antibody levels they were able to achieve *in vivo* were low, likely because the system they used contained only a secretory antibody lacking BCR function and a non-B cell promoter. While the theoretical maximum levels of antibody achieved with the Molecular Rheostat might be less than an ideal, natural, alternative-splicing system due to the fixed diversion of some antibody production capacity toward the expression of surface BCRs, the Molecular Rheostat system, by providing a membrane BCR, opens the possibility of harnessing the enormous numerical amplification power of B-cell clonal expansion following antigen encounter and the hugely upregulated synthetic and secretory capacity of plasma cells that arise as a result of B cell activation through the BCR. This will enable the provision of high levels of circulating anti-HIV broadly neutralizing antibodies by taking advantage of two very powerful amplification mechanisms of the natural immune system. In this regard, the results from the EU12 and OCI-Ly7 cell experiments are very promising – they show that so long as there are sufficient surface levels of BCR expression, the specific choice of 2A mutants is not that important in promoting B cell maturation or allowing B cell function, since both the “intermediate” and “maximum surface” contructs were able to promote B cell maturation and both “high” and “low” surface mutants were able to generate Ca++ signals to the B cells. This provides great flexibility in the choice of mutants to use.

In this study, we used two B-cell derived promoters, MH and EEK. In *in vitro* tests using cell lines, we found genes driven by these promoters to be highly expressed in B-lineage cells but only weakly in a non-B lineage cell line, 293T ([23] and unpublished data). The use of B cell specific promoters would enable high level expression from B cells, taking advantage of the natural clonal expansion and amplification process following encounter with HIV antigens, and provides a fairly natural way to express antibody genes. However, we believe that the system would still work even if the promoters were not completely B-cell specific and antibodies were secreted by some non-B lineage cells. If any antibodies were produced by non-B lineage cells due to non-specificity of the promoters, they would not inhibit anti-HIV B-cell development in the bone marrow because there B-cell development depends only on the presence of a functional BCR and not on high levels of antigens (which would actually inhibit B cell development). Minor non-specificity of the promoter giving rise to additional antibodies could actually enhance protection against HIV infection because they could provide a basal, constitutive level of protection against low amounts of virus, while the antigen-specific B cells would respond to the “breakthrough” viruses that surmounted the basal level of circulating antibodies. The Molecular Rheostat system thus provides a robust means to genetically program anti-HIV B cells.

In practice, the level of expression of a vector-delivered gene is a function of many factors, including, importantly, copy number variation and vector integration site effects as well as the choice of promoters. Some transduced cells will receive more copies of the transgene at more favorable chromosomal locations than others. With the Molecular Rheostat system, clones of cells with good surface expression will likely be preferentially selected to develop *in* *vivo* because the natural process of B cell development favors cells with good BCR expression. This will translate into the selection of cells that are also able to produce higher levels of secretory antibody because the ratio of surface to secreted antibody is fixed by the choice of the F2A mutant. For this reason, so long as only *one* F2A mutant is used in any given individual, the clones of cells with the highest gene expression levels (both surface expression and secretion) will be preferentially selected. The goal then is to choose the 2A mutant that maximizes secretion, as long as sufficient surface BCR expression is maintained so that antigen-specific B cells are able to develop from the bone marrow and respond to antigens. This could be done in practice by screening the different mutants of the Molecular Rheostat library in bone marrow transplant animal models and selecting the 2A mutant that yielded the highest antibody concentration while providing mature antigen-responsive B cells.

In summary, the flexibility of being able to use a range of mutants in the Molecular Rheostat library coupled with the choice of strong B cell promoters (such as EEK and MH) makes it a promising gene therapy approach to HIV prophylaxis. While we used the b12 antibody in this work, a “cocktail therapy” approach by using multiple anti-HIV broadly neutralizing antibodies in separate vectors could increase the spectrum of coverage of the system. With some slight modifications, this system of simultaneous BCR and IgG expression may also be used to manipulate B cells to target other antigens.

The principle of using a library of mutant 2A peptides as a Molecular Rheostat to mediate *tunable* simultaneous expression of two different proteins are not limited to B- cell programming, and may be applied to other contexts, such as the study of multi-domain transcription factors, or the engineering of other polyprotein expression systems, where a full-length protein and a partially truncated one are required at controllable stoichiometric amounts. In gene therapy, an important problem is to track cells that express a vector-delivered transgene–this is especially important in the event of an adverse reaction. A particularly interesting application of the Molecular Rheostat is to use it as a “flag” to mark cells that express an exogenous soluble protein, such as a soluble therapeutic antibody, by displaying a small amount of the transgenic antibody on the surface. A version of the Molecular Rheostat biased toward secretion with a small amount of surface expression can be used to connect the soluble antibody with a mutant IgM-transmembrane domain that mediates Igα- and Igβ-independent expression (e.g., the YS-VV mutant [38], [39]) or another membrane anchor (e.g. the GPI-anchor signal of LFA-3 [40]). Most of the antibody produced will be secreted, but a small amount of the full-length antibody-anchor fusion will be expressed on the cell surface. This provides a compact way to mark the transduced cells by displaying the transgenic antibody as a “flag” that can be easily recognized by either the antibody’s cognate antigen, or an anti-idiotypic antibody against the transgenic antibody. The cognate antigen or anti-idiotypic antibody coupled to a marker molecule or a toxin can then be used to track or eliminate the transgene-expressing cells. The Molecular Rheostat is thus a versatile tool for protein engineering and gene therapy.

## Supporting Information

Figure S1 **FACS histograms of surface IgG expression of OCI-Ly7 cells transduced with different Molecular Rheostat constructs.** Green: surface IgG expression from different mutant 2A peptides in the Molecular Rheostat Immunoglobulin genes. Red: control L+H contruct (secreted antibody only). The plots are arranged in the same rank order as that shown in Figure 3A.
(TIF)
