# Supplementary figures and images for: Use of Mutated Self-Cleaving 2A Peptides as a Molecular Rheostat to Direct Simultaneous Formation of Membrane and Secreted Anti-HIV Immunoglobulins
Source: PLoS One. 2012 Nov 28;7(11):e50438. doi: 10.1371/journal.pone.0050438 (PMC3508920; doi:10.1371/journal.pone.0050438)

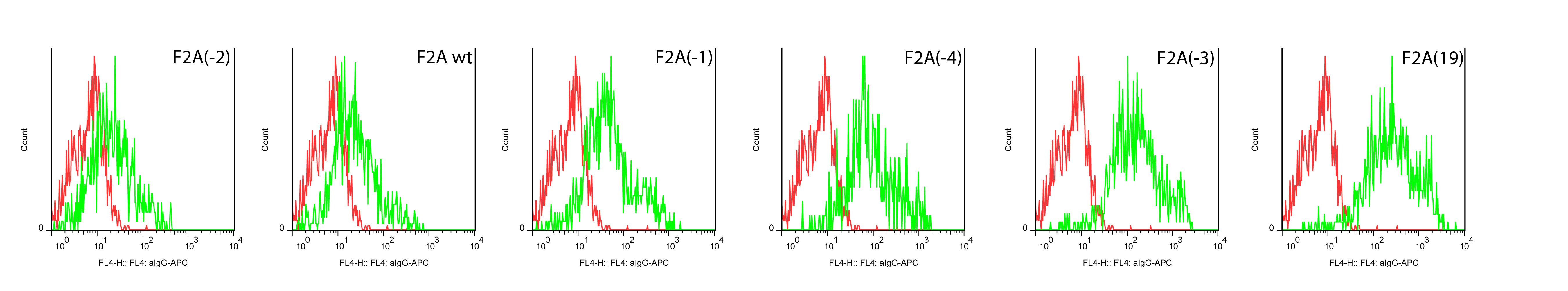

Supplement: Figure S1 — FACS histograms of surface IgG expression of OCI-Ly7 cells transduced with different Molecular Rheostat constructs. Green: surface IgG expression from different mutant 2A peptides in the Molecular Rheostat Immunoglobulin genes. Red: control L+H contruct (secreted antibody only). The plots are arranged in the same rank order as that shown in Figure 3A. (TIF) [file pone.0050438.s001.tif]
